# Efficacy of a fixed combination of 0.09 % xanthan gum/0.1 % chondroitin sulfate preservative free vs polyethylene glycol/propylene glycol in subjects with dry eye disease: a multicenter randomized controlled trial

**DOI:** 10.1186/s12886-016-0343-9

**Published:** 2016-09-20

**Authors:** Ana L. Pérez-Balbuena, Juan C. Ochoa-Tabares, Sandra Belalcazar-Rey, Cristian Urzúa-Salinas, Laura R. Saucedo-Rodríguez, Regina Velasco-Ramos, Raúl G. Suárez-Sánchez, Adolfo D. Rodríguez-Carrizalez, Aldo A. Oregón-Miranda

**Affiliations:** 1 Department of anterior segment, Asociación para evitar la ceguera en México, Hospital Dr. Luis Sánchez Bulnes, IAP, Ciudad de México, Mexico; 2 Cornea specialized attention, Private. Guadalajara, Jalisco, Mexico; 3 Department of Ophthalmology, Fundación Oftalmológica Nacional, Bogota, Colombia; 4 Department of Ophthalmology, Hospital del Salvador, Santiago, Chile; 5 Department of anterior segment, Antiguo Hospital Civil de Guadalajara Fray Antonio Alcalde, Guadalajara, Jalisco Mexico; 6 Department of anterior segment, Fundación Hospital Nuestra Señora de la Luz. IAP, Ciudad de México, Mexico; 7 Department of Ophthalmology, Instituto Médico de la Visión, Ciudad de México, Mexico; 8 Clinical Research Department, Laboratorios Sophia, SA de CV, Zapopan, Jalisco Mexico

**Keywords:** Xanthan gum, Chondroitin sulfate, Dry eye, Tear film, OSDI

## Abstract

**Background:**

Dry eye disease (DED) is multifactorial, affecting 5–34 % of the global adult population and reducing quality of life. The artificial tears or lubricants are the therapy most used for the treatment of DED, due to their low side effect profile, which attempt to modify the properties of the tear film. The aim of the present study was to evaluate the clinical efficacy of a fixed combination of xanthan gum and chondroitin sulfate preservative free on the ocular surface of patients with dry eye disease during 60 days of intervention.

**Methods:**

A phase III, double-blind, masked, controlled, multicenter, clinical trial of 148 subjects, randomized to either a fixed combination of xanthan gum 0.09 % and chondroitin sulfate 0.1 % (XG/CS) ophthalmic solution (*n* = 76) or a fixed combination of polyethylene glycol 400 0.4 % and propylene glycol 0.3 % (PEG/PG) (*n* = 72). Subjects self-dosed four times daily during 60 days. Follow-up was set on days 2, 7, 15, 30 and 60. Assessments of anterior/posterior segment ocular signs were performed. The outcome measures included Schirmer test, tear film break-up time and OSDI score. Security variables included intraocular pressure, lisamine green and fluorescein ocular surface stains.

**Results:**

The primary efficacy endpoints were similar between groups at baseline. After intervention time Schirmer test increased in both groups compared to baseline, XG/CS (6.4 ± 2.2 vs 11.0 ± 6.6; *p* = 0.002) and PEG/PG (6.5 ± 2.5 vs 10.5 ± 5.6; *p* = 0.019) respectively. Similar results were reported in the tear film break-up time in XG/CS (5.5 ± 2.1 vs 7.4 ± 2.9; *p* = 0.027) and PEG/PG (5.2 ± 2.0 vs 7.4 ± 2.7; *p* = 0.046) respectively. The OSDI score decreased to normal values in both groups, XG/CS (19.3 ± 7.4 vs 7.3 ± 5.9; *p* = 0.001) and PEG/PG (19.3 ± 7.5 vs 7.9 ± 8.2; *p* = 0.001) respectively. There was no significant difference between treatments for any parameter. Moreover, both groups decreased the presence of burning sensation, tearing, foreign body sensation, conjunctival hyperemia and photophobia. The adverse events were not related to the interventions.

**Conclusions:**

Xanthan gum/chondroitin sulfate preservative free showed similar clinical efficacy, evaluated with OSDI score, TBUT and Schirmer test compared to polyethylene glycol/propylene glycol in the treatment of dry eye disease.

**Trial registration:**

ClinicalTrials.gov: NCT01657253. Date of registration May 19, 2014.

## Background

The tear film is classically schemed as a three-phase emulsion, composed by an aqueous, a mucinous and a lipid layer; together have viscoelastic properties that allows them to adhere to the ocular surface in order to provide moisture, protection, nutrition and effects in optical quality. However, each layer have a particular function; the mucin layer, produced by goblet cells, plays an important role in protecting the ocular surface, stabilizing tears and acting as a gel-like barrier. The quality and quantity from the tear film is highly influenced by the conditions and abnormalities of the ocular surface [1–3]. Alterations to produce good quality of tears or a sufficient amount of tears involve abnormalities in the ocular surface [4]. Dry eye disease (DED) is multifactorial, affecting 5–34 % of the global adult population and reducing quality of life [5]. Altered mucin production can reduce tear film stability, increase osmolarity, and can to ignite an inflammation response that perpetuates a vicious circle of disease progression [6].

The artificial tears or lubricants are the therapy most used for the treatment of DED [7], due to their low side effect profile, which attempt to modify the properties of the tear film with the objective to increase their ocular residence time or restore the affected layer. However, this therapy must be preservative free due to adverse effects on ocular surface of the most preservatives used in Ophthalmology.

Xanthan gum is an exopolysaccharide with rheological properties that could provide stability and increase the residence time of the tear film on the ocular surface [8, 9]. Chondroitin sulfate used in ophthalmology is a Newtonian fluid that adheres readily to the surface epithelium and slows the evaporation of the aqueous layer [10–12]. These pharmacologic characteristics can to act in a synergistic way and reinforce the properties of tear film. Previously, Llamas-Moreno et al. [13] demonstrated that ophthalmic solution with XG/CS preservative free was effective in the treatment of dry eye disease decreasing OSDI score to normal values. However, due to a small sample there were not statistically differences in TBUT and Schirmer test compared with PEG/PG. In other hand, PEG/PG has showed its efficacy and safety in the treatment of DED due to restructures the tear film by forming a gel matrix that provides long-lasting protection. In this context, both lubricants can to increase the residence time of the tear film by different mechanisms. XG/CS preservative free is an option in the treatment of DED with the advantage of protects the ocular surface.

The aim of the study was to evaluate the safety and efficacy of the fixed combination of XG/CS preservative free in subjects diagnosed with mild to moderate dry eye disease in Latin American population compared with PEG/PG.

## Methods

A parallel, randomized, double blind, active-controlled, multicenter, clinical trial was designed to compare the efficacy of two ophthalmic solutions. The study was conducted across 7 investigative sites (5 in Mexico, 1 in Colombia and, 1 in Chile). An ethics committee in each center reviewed and approved the study. The clinical trial was conducted in accordance with Good Clinical Practice Standards (as described by the International Conference of Harmonisation) and Declaration of Helsinki. All patients provided written informed consent. The study was registered at ClinicalTrials.gov with the identifier number NCT01657253.

Inclusion criteria were patients (aged >18 years) with best corrected visual acuity < 0.6 logMAR or better in both eyes, mild to moderate dry eye disease based on Ocular Surface Disease Index (OSDI) score between 12–45 and without active ocular disease and no use of topical ocular drops within approximately 24 h before screening. Exclusion criteria were patients with autoimmune disease (eg, Sjögren syndrome, etc.) Meiboian gland dysfunction, blepharitis, corneal dystrophy, eyelid malformations, history of eye surgery within 3 months before baseline, intolerance or hypersensitivity to any component of study treatments, contact lens users, participation in an investigational drug or device study < 60 days before screening, ocular o systemic infections or conditions (eg, epithelial herpes simplex keratitis, vaccinia, varicella or mycobacterial infection; fungal disease; iritis) that preclude safe administration of study treatment, and patients that were pregnant, at risk for pregnancy without birth control treatment, or breastfeeding. Written informed consent was received from each subject prior to any study related procedure. Patients were randomly allocated 1:1 to received XG/CS (Xiel ofteno®, Sophia Laboratories, SA de CV, Zapopan, Jalisco, Mexico) or PEG/PG (Systane®, Alcon Laboratories, Inc, Fort Worth, TX, USA) using random numbers software. Baseline data including demographics, relevant medical and ocular history, and concomitant medications were noted at visit 1 (Day 0). Subjects instilled one drop of study drug topically in the inferior conjunctival sac of both eyes four times daily. The compliance was evaluated according this formula: weight of the bottle after intervention × 100 / weight of the bottle before intervention was started. An adequate compliance was considered >80 %. The compliance was evaluated in 1 bottle each 30 days. The final compliance was determined using the mean of both bottles. Moreover, the pharmacist verified the register of eyedrop instillation from patient diary. Investigators were masked to the study medication. Because the active control bottle (Systane) was visibly different than the investigational bottle, a designee at each study site, other than the investigator, was responsible for the dispensing study treatment. Attempts were made to mask the subjects by removing commercial labeling, replacing it with identical investigational labels and packaging in identical kit boxes and were separated during the evaluations. Patients were evaluated during six study visits: Visit 2 (Day 2 ± 1), Visit 3 (Day 7 ± 1), Visit 4 (Day 15 ± 1), Visit 5 (Day 30 ± 1) and, Visit 6 (Day 60 ± 1) after randomization. Clinical assessments during baseline and the final visit consisted of intraocular pressure (IOP), using a calibrated Goldman applanation tonometer, tear break-up time (TBUT), Schirmer I test with anesthesia, and indirect ophthalmoscopy. Slit lamp assessment (biomicroscopy) and fluorescein and green lisamine stain were performed. Safety assessments included adverse events (AEs) and ocular tolerability (burning sensation, tearing, foreign body sensation, conjunctival hyperemia and photophobia).

The primary efficacy endpoints were the increase from baseline in mean Schirmer test, TBUT and, a reduction of OSDI score at visit 6 (Day 60). The safety endpoint was the incidence of ocular and systemic AEs and their severity and relationship to the study drug.

### Statistical analysis

The results are presented in mean and standard deviation. Kolmogorov-Smirnov test was made to know the normal distribution of data. We considered both eyes for statistical analysis. An intent-to-treat analysis was performed. Inter-eye correlation was made within the same subject. The mean of IOP, Schirmer test, TBUT and OSDI score were compared using paired two-sided t tests. Ocular signs and symptoms were summarized using proportions and were analyzed with the chi-square method. In all analyses, a *p*- value of < 0.05 (two-tailed) was considered statistically significant. Adverse reactions were evaluated using the collection method. All statistical analyses were conducted using SPSS software (IBM Corporation. Armonk, NY, USA) version 19.

## Results

**Fig. 1:**
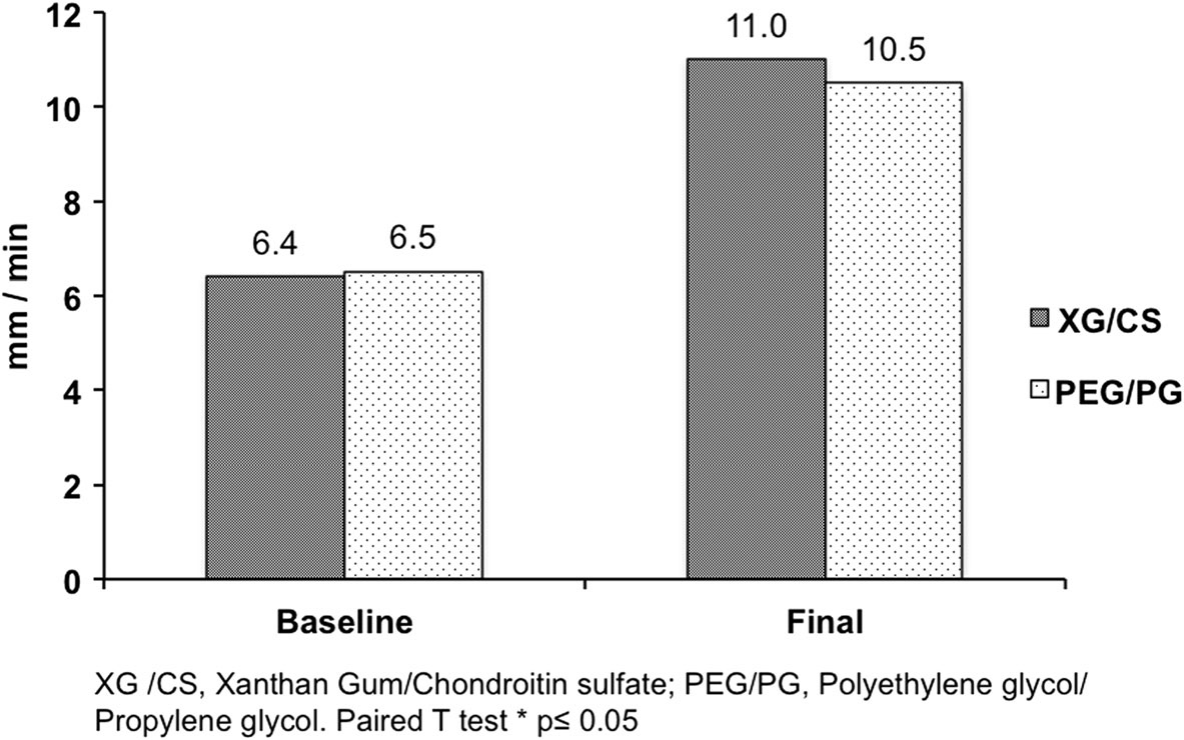
Schirmer test before and after intervention in both groups. Schirmer test after intervention showed statistically significant difference compared to baseline in XG/CS (*p* = 0.002) and PEG/PG (*p* = 0.019) but not between them

**Fig. 2:**
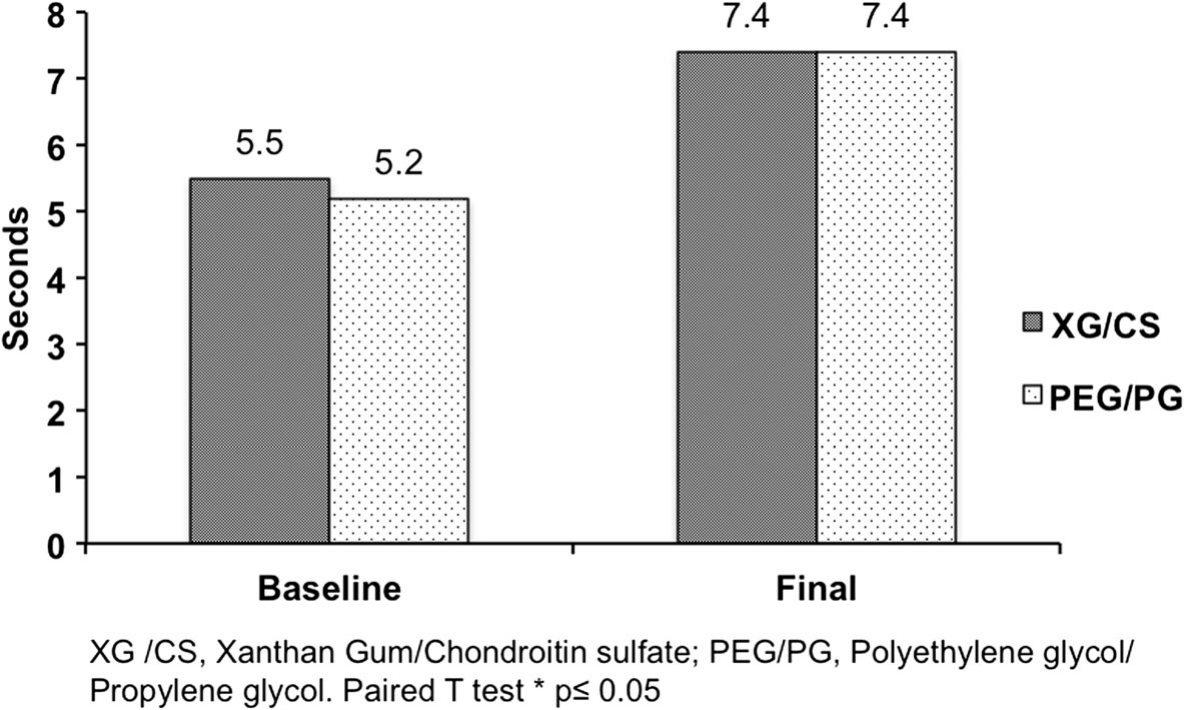
TBUT before and after intervention in both groups. TBUT after intervention showed statistically significant difference compared to baseline in XG/CS (*p* = 0.027) and PEG/PG (*p* = 0.046) but not between them

**Fig. 3:**
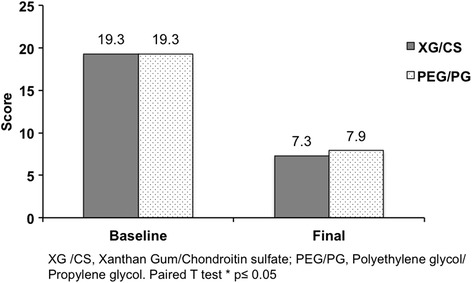
OSDI score before and after intervention in both groups. OSDI score after intervention showed statistically significant difference compared to baseline in XG/CS (*p* = 0.001) and PEG/PG (*p* = 0.001) but not between them

The treatment groups were comparable regarding to demographics and baseline characteristics (Table 1). Of the 190 subjects screened, 183 subjects were randomized (*n* = 93, XG/CS; *n* = 90 PEG/PG) and 148 subjects completed the study (*n* = 76, XG/CS; *n* = 72, PEG/PG) without statistical difference. According to Kolmogorov-Smirnov test the data have a normal distribution. The primary efficacy endpoints were similar between groups at baseline. After intervention time, Schirmer test and TBUT increased in both groups compared with baseline (Figs. 1 and 2). The OSDI score decreased to normal values in both groups (Fig. 3). There were not significance differences to compare groups. Moreover, both groups decreased the presence of burning sensation, XG/CS (77 to 48 %; *p* = 0.007), PEG/PG (72 to 34 %; *p* = 0.006); tearing, XG/CS (50 to 23 %; *p* = 0.027), PEG/PG (28 to 17 %; *p* = 0.006); foreign body sensation, XG/CS (79 to 29 %; *p* = 0.027), PEG/PG (80 to 28 %; *p* = 0.027); conjunctival hyperemia XG/CS (59 to 28 %; *p* = 0.007), PEG/PG (73 to 35 %; *p* = 0.007); and photophobia, XG/CS (58 to 31 %; *p* = 0.027), PEG/PG (80 to 29 %; *p* = 0.027). There were no alterations in corneal stains. The adverse events were not related to the interventions.

**Table 1:** Demographics and clinical characteristics in three Latin American populations at baseline

	Chile			Colombia			Mexico		
	XG/CS *n* = 32	PEG/PG *n* = 24	p^a^	XG/CS *n* = 26	PEG/PG *n* = 20	p^a^	XG/CS *n* = 94	PEG/PG *n* = 100	p^b^
Age, years									
Mean (SD)	38.6 (13.6)	35.3 (11.8)	0.336	52.9 (13.7)	52.7 (12.4)	0.824	50.8 (16.7)	48.3 (13.5)	0.273
Gender, N (%)									
Female	13 (81)	8 (67)	0.212	10 (77)	8 (80)	0.802	20 (77)	16 (80)	0.958
Male	3 (19)	4 (33)		3 (23)	2 (20)		6 (23)	4 (20)	
IOP, mmHg									
Mean (SD)	14.2 (2.5)	13.7 (1.2)	0.471	12.5 (2.3)	13.2 (1.6)	0.535	12.8 (2.5)	13.1 (2.4)	0.201
Schirmer test, mm/min									
Mean (SD)	6.2 (2.4)	7.1 (2.6)	0.114	6.0 (2.8)	6.3 (2.8)	0.674	6.8 (1.8)	6.2 (2.4)	0.233
TBUT, sec									
Mean (SD)	5.5 (1.3)	6.0 (1.8)	0.292	4.9 (2.0)	4.5 (2.0)	0.487	5.8 (2.2)	4.9 (1.9)	0.073
OSDI, score									
Mean (SD)	18.5 (6.6)	20.0 (9.2)	0.714	19.8 (7.9)	15.4 (2.4)	0.070	19.4 (6.9)	20.6 (7.4)	0.234

*XG/CS* Xanthan gum/Chondroitin sulfate, *PEG/PG* Polyethylene glycol/Propylene glycol, *IOP* Intraocular pressure, *TBUT* Tear break-up time, *OSDI* Ocular Surface Disease Index. ^a^Wilcoxon T test. ^b^Paired T test. *p* ≤ 0.05. *n* = total eyes

## Discussion

The ideal lubricant should be able to restore the affected component of tear film regardless etiology, with less frequency of instillation, and above all non adverse effects on the ocular surface [14–16]. A main limitation of artificial tears available is the short duration of symptom relief due to restricted precorneal residence time.

Xanthan gum is a polymer with mucoadhesive properties that creates synergic interactions with mucin molecules playing an important role in the formation of the mucus layer of the tear film [17]. In addition, like others polysaccharides, has a high affinity for water and is able to increase viscosity [18, 19]. Regardless of this characteristic, XG alone or in combination has been tested poorly in clinical practice. The pharmacology profile of xanthan gum in combination with sodium hyaluronate has been explored in the treatment of corneal abrasions showing efficacy and protection of the ocular surface after 7 days of intervention. Faraldi et al., concluded the importance of hydration and protection of the cornea due to water binding and mucoadhesivity properties provided by XG and sodium hyaluronate [20]. Moreover Llamas-Moreno et al. demonstrated the clinical efficacy of XG/CS in patients with similar characteristics, improving OSDI score compared to PEG/PH-HP Guar. This significant change in OSDI score reach to normal values, showing the importance of the increase in the residence time of a lubricant. However not significant changes either TBUT or Schirmer test were reported between groups.

In other hand, PEG/PG works by binding to the hydrophobic exposed areas of the epithelial cells, attaching a protective HP-Guar tear-gel matrix that helps restore the ocular surface. In a review, PEG/PG has showed in several clinical trials its efficacy and safety studies compared with different lubricants.

Clinical symptoms, TBUT, Schirmer test, and OSDI score are the most sensitive and specific tests performed to evaluate the severity of DES [7, 21]. Changes in these parameters help to understand the clinical efficacy of a treatment. In the current study, the group that received XG/CS improved its results in all tests above mentioned. However, there were no differences when was compared with PEG/PG after 60 days of intervention.

These results could be due to mucoadhesivity properties of xanthan gum, which increased the corneal residence time of the tear film. On the other hand, CS has moisturizing properties delaying evaporation of aqueous layer due to its effect like-coat on the ocular surface [22–24].

In addition to rheological properties above described, both, XG and CS act on different pathophysiological points involved in DED development. Xanthan gum is rich in OH-groups allowing it to react with reactive oxygen species and preventing oxidative stress damage implicated as a possible pathogenic cause of DED [25]. Meanwhile, CS modulates the inflammatory response mediated by cytokines [23].

One limitation of this study is the lack of measurement of some inflammatory and oxidative markers described in the pathogenesis of DED [26, 27].

## Conclusions

Xanthan gum/chondroitin sulfate preservative free showed similar clinical efficacy, evaluated with OSDI score, TBUT and Schirmer test compared to polyethylene glycol/propylene glycol in the treatment of dry eye disease.

## Abbreviations

AEs: Adverse events
CS: Chondroitin sulfate
DED: Dry eye disease
IOP: Intraocular pressure
OSDI: Ocular surface disease index
PEG: Polyethylene glycol
PG: Propylene glycol
TBUT: Tear film break-up time
XG: Xanthan gum
